# Extensive Medullary Cancer of the Antrum

**Published:** 1861-03

**Authors:** 


Extensive Medullary Cancer of the Antrum—Removal of the Tumor, with the Superior Maxillary Bone and adjacent Bony and Soft Parts.—This is in an elderly female from the interior of the State. The affection is of some eighteen months' standing. The tumor occupies the antrum of the upper jaw on the right side, and by careful examination of some of the matter under the microscope, it has been found to be soft cancer, or fungus *hæmatodes.*

These tumors of the antrum are seen in persons of all ages, probably more frequently in advanced life, though Dr. Pancoast has seen them in children, in babes, and operated on them.

They begin, usually, in the lining membrane of the antrum, a mucous membrane, over the outer surface of which, between it and the bone, runs a plexus of nerves, many of which go to the incisor teeth.

In elderly people this antrum is a large cavity, and being bounded inferiorly by the alveolar ridge, which disappears when the teeth disappear or are removed, there remains often but a very thin structure, indeed, below the lower wall of the antrum. If there is a chronic inflammation of this membrane, giving rise to deposits, it will, at first, be accompanied with pain, by involving the plexus of nerves over the outer surface. The first intimations of this disease are excessive pains in the cheek bone, coming on periodically, for some reason that we do not understand.

In the present case, these pains were thought to be toothache, and one tooth after another was extracted, until all the teeth in the upper jaw were removed. But the pain continued. After a while, an opening took place in the mouth, near where one tooth had been drawn. Sometimes, indeed, these diseases of the antrum arise in consequence of disease of the inner lining or socket of the tooth.

From this opening there is a sanious discharge, excessively offensive, and passing into the nostril. There is constant and continuous pain, and the disease has extended high on the jaw, and very probably the absorbent vessels have gradually taken away the bone, so that there may be no bony wall at all. In addition to this, the disease has involved the masseter muscle.

There is anchylosis of the jaw, and the patient's mouth constantly open. It is one year since all the teeth have been removed.

The only chance to cure this case is to remove the whole diseased mass.

Whenever the disease has not surmounted the boundaries of the cavity, there is a good chance of effecting a cure ; for the operation can be made entirely beyond the boundary of the disease ; but when it has involved the periosteum and is contiguous with the substance of the cheek, it almost always returns and finally destroys the patient.

Various methods have been advised for performing the operation ; a favorite one is a V shaped incision. Another is sometimes made in the shape of the letter N. In the present case, where there will be a difficulty in removing all the diseased portions of tissue, the object will be best accomplished by means of a V shaped incision, with the apex down and the base upwards.

The operation was then performed by making an incision in the vertical line, across the face, over the orbicularis muscle, and over the angle of the mouth, cutting the facial artery; the other incision was made from near the inner canthus of the eye to that part of the lip over the canine tooth. When the parts were exposed, the orbital process of the malar bone was divided. After separating the nasal process of the upper maxillary bone, the roof of the mouth was divided with a chain saw, and the palate bone separated from the velum. During the operation, the masseter muscle was found to be diseased, and part was cut away; the malar bone and part of the zygomatic process, belonging to the temporal bone, had to be taken away. Many of the branches of the internal maxillary artery were cut across, but the main trunk was not interfered with. The disease involving the coronoid process of the lower jaw, a part was removed, cutting across the tendon of the temporal muscle.

The infiltration of cancerous matter extended into the internal pterygoid muscle, and its origin in the fascia between the pterygoid plates in the pterygoid process of the sphenoid bone. This was scraped out and the hot iron applied to the parts.

Jan. 5th. The patient has had no fever, and rested pretty well at night, though a little uneasy.

The wound united everywhere by the first intention.

The periosteum which lines the lower portion of the orbital cavity, having been taken away on account of its morbid appearance, this gave rise to some inflammation, which produced œdema of the lid.

The sutures have been taken away and a pad has been placed in the mouth to fill out the cheek, until nature shall have overcome the necessity for such a resort by the organization of plastic matter, which will be effused from the surface of the wounds made in the operation.

The patient has been allowed beef tea and milk punch. It is important to bear in mind that in these cases supporting treatment is generally necessary.

All the lines of incision about the face were curved, not only because the deformity would be thereby diminished, but also because there is less power of the muscles to draw open the sides of the wounds, than if the parts had been divided in straight lines. Another object gained is, that by these means every portion of the skin can be properly adjusted, and the natural position of the two flaps restored, which is necessary, in order to avoid paralysis of the face, which might otherwise ensue; for, in these operations the nerves of the face are necessarily divided; but if they are very nicely approximated, there will be little or no interruption in the transmission of the nervous influence.—*Medical and Surgical Reporter.*

